# Dietary Intake, Nutritional Status, and Sarcopenia in Patients with Liver Cirrhosis: a Cross-Sectional Study

**DOI:** 10.34172/mejdd.3338

**Published:** 2026-01-31

**Authors:** Elham Sobhrakhshankhah, Farhad Zamani, Parvin Hassanzadeh, Soghra Karimi, Amir Hossein Faraji, Seyed Yahya Zarghami, Naemeh Kolahdoozan, Mohana Eskandari, Azam Doustmohammadian

**Affiliations:** ^1^Gastrointestinal and Liver Diseases Research Center, Iran University of Medical Sciences, Tehran, Iran; ^2^Department of Nutrition, Firoozgar General Hospital, Iran University of Medical Sciences, Tehran, Iran; ^3^Division of HPB Surgery & Abdominal Organ Transplantation, Firoozgar Hospital, Iran University of Medical Sciences, Tehran, Iran; ^4^Department of Internal Medicine, School of Medicine, Firoozgar General Hospital, Iran University of Medical Sciences, Tehran, Iran

**Keywords:** Sarcopenia, Nutritional status, Liver cirrhosis

## Abstract

**Introduction:**

Liver cirrhosis is associated with significant nutritional challenges, including malnutrition and sarcopenia, which impact clinical outcomes. This study aimed to evaluate the nutritional status, dietary intake, and sarcopenia in patients with liver cirrhosis and to explore potential sex-specific differences.

**Methods:**

In this cross-sectional study, 92 adult patients with clinically confirmed cirrhosis were recruited from a tertiary referral center between February 2024 and January 2025. Nutritional status was assessed using anthropometric measurements. A validated 168-item Food Frequency Questionnaire (FFQ) was used to estimate habitual energy intake, macronutrient intake, and daily servings of major food groups. Sarcopenia was defined according to the Asian Working Group for Sarcopenia (AWGS 2019), incorporating handgrip strength, mid-upper arm circumference (MUAC), and six-minute walk distance.

**Results:**

Among 92 patients with liver cirrhosis (mean age 49.1±13.5 years; 57.6% men), sarcopenia was present in 66.3% and was more frequent among unmarried individuals (*P*=0.046). Only 38% met vegetable intake recommendations, 56.5% achieved plant-based protein targets, and 20.7% reached the dairy intake threshold. A comparative analysis of dietary intake revealed no statistically significant differences in total energy, total protein, or most micronutrients between sarcopenic and non-sarcopenic patients.

**Conclusion:**

Sarcopenia remains highly prevalent among patients with liver cirrhosis, alongside notable deficiencies in vegetable and dairy consumption. The interplay among social, anthropometric, and functional factors underscores the need for integrated, multidisciplinary strategies to improve outcomes for this vulnerable population.

## Introduction

 Liver cirrhosis remains a major global health challenge, accounting for more than 1.3 million deaths annually, a figure projected to rise in tandem with the increasing burden of metabolic-associated liver disease and alcohol-related hepatic injury.^1^ While cirrhosis has traditionally been understood in terms of liver failure and high blood pressure in the portal vein, it is now seen as a condition that affects multiple systems in the body. Serious changes in metabolism and nutrition characterize this. Some of the most significant issues are malnutrition, frailty, and sarcopenia. Sarcopenia is the gradual and severe loss of skeletal muscle mass, strength, and physical function. Nearly half of the people with advanced liver disease experience these conditions. They have been linked to worse clinical outcomes, including higher hospitalization rates, increased mortality after transplant, and a significantly lower quality of life.^2^

 Recent advances have shifted clinical focus beyond traditional hepatic endpoints to encompass these extrahepatic complications as critical determinants of prognosis.^3^ Sarcopenia and related syndromes are not only highly prevalent, affecting approximately 30–50% of patients with cirrhosis, but also represent modifiable risk factors that may be amenable to targeted nutritional and rehabilitative interventions.^4,5^

 Among these, sarcopenia has gained particular importance. Sarcopenia involves a gradual and widespread decline in muscle mass and strength. It is driven by several factors, including chronic inflammation, high ammonia levels, hormonal imbalances, a lack of physical activity, and insufficient or improper nutrient intake.^6^ This accelerated muscle wasting often develops insidiously and may precede overt hepatic complications, making it a critical but under-recognized therapeutic target in early and advanced liver disease alike.

 Nutritional assessment in cirrhosis presents unique challenges due to confounding factors such as fluid retention, altered metabolism, and frequent underreporting of intake. Nevertheless, growing evidence suggests that poor dietary quality and inadequate energy and protein intake are key contributors to sarcopenia and malnutrition in this population.^7^ While most clinical guidelines recommend protein intakes of 1.2–1.5 g/kg/day and multiple small meals, particularly late-evening snacks, data from real-world populations suggest that many patients fall short of these targets.^8,9^ Moreover, the specific nutrient gaps and overall nutritional status of patients with cirrhosis, especially in non-Western contexts, are still not well understood. Another emerging but underexplored aspect is the role of sex in influencing the risk and severity of sarcopenia.^10^ Biological differences in body composition, hormonal regulation, and nutrient metabolism, along with social and behavioral differences in eating habits, may lead to different vulnerabilities to muscle loss between men and women.^11^ However, few studies have examined these differences, and data from the Middle Eastern population on these assessments remain scarce.^12^

 To address these gaps, we conducted a cross-sectional study of adult patients with clinically confirmed liver cirrhosis. We aimed to evaluate their nutritional status, typical dietary intake, and the presence and severity of sarcopenia. Furthermore, we hoped to explore potential sex-specific patterns in nutritional deficiencies and outcomes related to sarcopenia. By integrating objective anthropometric assessments, validated dietary instruments, and internationally accepted diagnostic criteria for sarcopenia, this study aimed to provide a nuanced, sex-stratified understanding of nutritional and functional impairments in cirrhosis, a crucial step toward more personalized and equitable care for this vulnerable population.

## Materials and Methods

###  Study Overview And Clinical Setting

 This observational, cross-sectional study was conducted from February 2024 to January 2025 at Firouzgar Hospital, a major academic and tertiary referral center affiliated with Iran University of Medical Sciences (IUMS) in Tehran. The study population comprised adult patients (aged ≥ 18 years) with clinically verified cirrhosis of diverse etiologies and disease severities, recruited consecutively from both the liver transplantation unit and the hepatology outpatient clinic.

###  Patient Enrollment And Eligibility Criteria

 Eligible patients were identified and enrolled consecutively during routine clinical encounters. Diagnosis of cirrhosis relies on a combination of clinical history, physical examination findings, lab results, imaging outcomes, and liver biopsy when necessary. We obtained written informed consent from all participants, who also demonstrated adequate cognitive and physical abilities to participate in the study. Exclusion criteria included severe systemic health issues that could affect the diagnosis of sarcopenia. These conditions included advanced chronic obstructive pulmonary disease, heart failure, chronic kidney disease, and active cancer. We also excluded individuals with cognitive impairments that would have hindered their ability to provide informed consent. We estimated the sample size based on previously published data on handgrip strength in cirrhotic populations. This data showed a standard deviation of 3.52 kg and a mean of 16.9 kg (13). Assuming a two-sided alpha of 0.01 and a precision of 1.0, the minimum sample size was calculated as 82 participants. To account for possible dropouts and preserve statistical power, a final sample of 100 patients was enrolled.

###  Clinical Evaluation And Data Acquisition

####  Sociodemographic and Medical Profile

 Structured interviews were conducted using a pretested questionnaire to capture sociodemographic characteristics (age, sex, education, employment status, marital status, and income), disease history (etiology of cirrhosis, medication use, comorbidities, smoking status), and clinical severity. Cirrhosis severity was established through the Child–Pugh.^14^ Child–Pugh scores incorporated ascites, hepatic encephalopathy severity, and laboratory values for albumin, bilirubin, and prothrombin time. Participants were then stratified into classes A (mild), B (moderate), or C (severe) according to disease severity.

####  Anthropometric Assessments

 Anthropometric indices were measured using standardized techniques. Body weight was recorded to the nearest 100 g using a calibrated Seca digital scale. Participants were barefoot and wore light clothing. Standing height was measured to the nearest 0.1 cm using a stadiometer. Waist circumference was measured halfway between the lowest rib and the iliac crest at the end of expiration. The hip circumference was measured at the level of the greater trochanters. Central obesity was defined as a waist circumference greater than 102 cm for men and 88 cm for women, according to ATP-III sex-specific cutoffs.^15^ Mid-upper arm circumference (MUAC) was measured at the midpoint between the acromion and olecranon on the non-dominant arm.

####  Sarcopenia Diagnosis And Functional Assessment

 Sarcopenia was defined according to the 2019 consensus criteria of the Asian Working Group for Sarcopenia (AWGS 2019) ^16^, requiring evidence of reduced skeletal muscle mass accompanied by either diminished strength or physical performance. Severe sarcopenia was diagnosed in the presence of concurrent deficits across all three domains.

 Skeletal muscle mass was estimated using MUAC, measured with a non-elastic tape to the nearest 0.1 cm. Sex-specific thresholds for low muscle mass were defined as ≤ 28 cm in men and ≤ 27 cm in women, in line with EWGSOP2 guidelines.^17^

 Handgrip strength (HGS) was assessed using a Jamar dynamometer (Model 14192-709E). Participants performed three trials using their dominant hand with 30-second rest intervals. The highest value recorded was used for analysis. Thresholds for low muscle strength were < 27 kg for men and < 16 kg for women.^18^

 Functional capacity was assessed using the 6-minute walk test (6MWT). It took place in a 30-meter indoor corridor, following international guidelines.^19^ A total walking distance < 250 meters was used to define impaired physical performance.^20^

####  Physical Activity Evaluation

 Physical activity was assessed using the Short Form of the International Physical Activity Questionnaire (IPAQ-SF).^21^ Participants reported how often and for how long they engaged in physical activity in various areas, including work, transportation, household tasks, and leisure activities, over the past 7 days. We calculated total physical activity in MET-minutes per week and categorized it as low, moderate, or high.

####  Dietary Intake Assessment

 Usual dietary intake over the previous year was assessed using a validated 168-item semi-quantitative Food Frequency Questionnaire (FFQ).^22^ Nutrient intake was calculated utilizing Nutritionist IV software, primarily based on the USDA Food Composition Table. For traditional Iranian items not listed in the USDA database, data were sourced from the Iranian Food Composition Table.

 Key dietary variables were derived, encompassing total daily energy and macronutrient intake, protein intake relative to body weight (g/kg), and daily consumption of major food groups based on standard portion sizes. Adherence to Iranian dietary guidelines was assessed for specific food categories, including fruits ( ≥ 2 servings/day), vegetables ( ≥ 3 servings/day), dairy products ( ≥ 1 serving/day), animal-based protein ( ≥ 1 serving/day), and plant-based protein ( ≥ 1 serving/day) ^23^ (see Supplementary Table 1). Additionally, energy and protein deficits were calculated by comparing each patient’s actual dietary intake against the clinical recommendations (35 kcal/kg and 1.2 g/kg, respectively) established by the EASL Clinical Practice Guidelines for nutrition in chronic liver disease.^24^

####  Statistical Analysis

 All statistical analyses were performed using IBM SPSS Statistics (version 26.0). Descriptive statistics were reported as mean ± standard deviations (SD) or medians with interquartile ranges (IQR). Categorical variables were shown as frequencies and percentages. Normality was checked using the Kolmogorov-Smirnov test. Between-group comparisons of dietary intake (sarcopenic vs non-sarcopenic and male vs female) were performed for total energy, macronutrients, and micronutrients using independent t-tests or Mann-Whitney U tests as appropriate. For categorical data, Chi-square tests or Fisher’s exact tests were used. A P value of 0.05 or less was considered statistically significant.

## Results

 Among the 92 participants, a substantial proportion of women (66.7%) were unemployed, whereas only about 10% were retired or employed in the formal sector. The prevalence of sarcopenia was higher in women (76.9%) than in men (58.5%); however, this difference was close to statistical significance, just short of the usual threshold (*P* = 0.065, Table 1).

**Table 1:** Comparison of demographics, anthropometry, and clinical characteristics between men and women

**Variable**	**Men (n=53) **	**Women (n=39)**	***P*** **value**^e^
Age (years)	48.85 ± 13.09	49.43 ± 14.09	0.837
Occupation, n (%)			
Unemployed	1 (1.9)	26 (66.7)	**<0.001**
Employee/Retired	18 (34.0)	4 (10.3)	
Self-employed	34 (64.2)	9 (23.1)	
Economic status, n (%)			
Poor	22 (41.5)	16 (41.0)	0.457
Moderate	24 (45.3)	21 (53.8)	
Good	7 (13.2)	2 (5.1)	
Marital status, n (%)			
Single/Divorced/Widowed	12 (22.6)	8 (20.5)	0.807
Married	41 (77.4)	31 (33.7)	
Education level, n (%)			
Illiterate	5 (9.4)	7 (17.9)	0.488
High school diploma or lower	39 (73.6)	26 (66.7)	
University degree	9 (17.0)	6 (15.4)	
Physical activity (MET)^a^, n (%)			
Low	38 (71.7)	34 (87.2)	0.075
Moderate to high	15 (28.3)	5 (12.8)	
Anthropometric indices			
Body Mass Index (BMI)	24.87 ± 3.98	24.72 ± 3.96	0.861
Waist circumference (cm)	95.68 ± 13.11	89.03 ± 9.46	0.008
Arm circumference (cm)	28.40 ± 3.89	26.92 ± 3.45	0.062
Calf circumference (cm)	36.05 ± 3.99	34.73 ± 7.95	0.303
Duration of cirrhosis (months), Median (IQR)	15 (31.0)	24 (56)	0.968
Child–Pugh classification, n (%)			
A + B	49 (92.5)	36 (92.3)	1.00
C	4 (7.5)	3 (7.7)	
Hospitalization			
No	6 (11.3)	9 (23.1)	0.131
Yes	47 (88.7)	30 (76.9)	
Presence of comorbidities, n (%)			
No	31 (58.5)	28 (71.8)	0.189
Yes	22 (41.5)	11 (28.2)	
Prevalence of sarcopenia and its components			
Sarcopenia, n (%)	31 (58.5)	30 (76.9)	0.065
Low muscle strength ^b^, n (%)	22 (41.5)	12 (30.8)	0.292
Low muscle function ^c^, n (%)	46 (86.8)	34 (87.2)	0.957
Low muscle mass ^d^, n (%)	26 (49.1)	22 (56.4)	0.485

Data were presented as mean ± SD and median (range) or as n of patients and percentages (%). **a. **Low levels of physical activity (PA): (PA)over the past 7 days < 600 (Met-Min/Week), moderate levels of (PA): (600-3000 MET- min/week) and high levels of (PA): ( > 3000 MET-min/week) due to the low percentage (2.2%) in the high levels of (PA), we combined the two levels of moderate and high to moderate to high (PA). **b. **Low muscle strength < 27 kg for men, and < 16 kg for women. **c. **A 6MWD of less than 400 m represents low muscle function. **d. **Lowmuscle mass: upper arm circumference < 27 cm for women and < 28 cm for men. **e.** Bolded values are significant at the < 0.05 level. Bold numbers indicate significance.

 Dietary intake exhibited significant sex-based disparities in patients with liver cirrhosis, with male patients consistently demonstrating higher median consumption across most nutritional components (Table 2). Men reported substantially greater absolute energy intake (3226.7 vs 2225.5 kcal/day; *P* = 0.010) and protein consumption (122.4 vs 77.6 g/day; *P* = 0.008), though these differences were attenuated when normalized to body weight. Similarly, carbohydrate intake was significantly elevated in men (417.0 vs 389.9 g/day; *P* = 0.007). This pattern extended to multiple micronutrients, with men showing significantly higher intake of potassium (*P* = 0.019), phosphorus (*P* = 0.006), magnesium (*P* = 0.008), iron (*P* = 0.034), and zinc (*P* = 0.010), along with several vitamins including E (*P* = 0.038), B₁ (*P* = 0.004), B₂ (*P* = 0.021), B₆ (*P* = 0.003), C (*P* = 0.010), and folic acid (*P* = 0.002).

**Table 2:** Comparison of dietary and nutrient intake between men and women

**Diet components**	**Men (n=53)**	**Women (n=39)**	***P*** **value**^e^
Energy intake (Kcal/day)	3226.7 (1860.6)	2225.5 (1420.9)	**0.010**
Energy intake (kcal/kg/day)	40.1 (28.5)	33.9 (23.6)	0.405
Energy deficit	-361.6 (2049.4)	78.8 (1547.1)	0.391
Protein intake (g/day)	122.4 (75.2)	77.6 (54.6)	**0.008**
Protein intake (g/kg/day)	1.5 (1.3)	1.3 (0.7)	0.405
Protein deficit	-28.3 (92.5)	-7.7 (48.3)	0.341
Fat intake (g/day)	90.8 (57.6)	64.7 (47.9)	0.051
Carbohydrates (g/day)	417.0 (269.9)	389.9 (209.6)	**0.007**
Saturated fatty acids (g/day)	29.3 (17.7)	22.1 (16.4)	0.077
Monounsaturated fatty acids (g/day)	31.4 (19.6)	21.9 (18.5)	**0.041**
Polyunsaturated fatty acids (g/day)	18.0 (11.3)	13.3 (12.0)	0.084
Cholesterol (mg/day)	343.2 (352.4)	220.8 (184.4)	**0.006**
Dietary fiber (g/day)	4.7 (4.0)	3.3 (4.3)	0.107
Sodium (mg/day)	4042.1 (2977.3)	3893.8 (2596.1)	0.819
Potassium (mg/day)	5281.9 (2763.7)	4391.7 (1902.4)	**0.019**
Calcium (mg/day)	1396.3 (961.6)	1026.3 (773.3)	0.054
Phosphorus (mg/day)	2045.9 (1221.4)	1504.0 (870.3)	**0.006**
Magnesium (mg/day)	545.9 (350.0)	395.4 (244.2)	**0.008**
Iron (mg/day)	34.3 (26.5)	26.1 (19.2)	**0.034**
Zinc (mg/day)	18.1 (8.4)	12.2 (7.7)	**0.010**
Vitamin A (μg/day)	724.5 (568.2)	570.4 (457.5)	0.155
Vitamin E (mg/day)	12.8 (9.9)	9.9 (7.8)	**0.038**
Vitamin B1 (mg/day)	2.5 (1.6)	1.8 (1.0)	**0.004**
Vitamin B2 (mg/day)	2.8 (1.8)	1.9 (1.0)	**0.021**
Vitamin B6 (mg/day)	2.8 (1.5)	2.1 (1.2)	**0.003**
Vitamin B12 (mg/day)	5.6 (4.4)	4.1 (4.3)	0.089
Vitamin C (mg/day)	221.4 (166.2)	149.5 (90.4)	**0.010**
Folic acid (μg/day)	778.7 (455.2)	493.8 (375.3)	**0.002**

Data were presented as median (IQR). Bolded values ​​are significant at the < 0.05 level. Bolded values are significant at the < 0.05 level

 Primary sclerosing cholangitis and Nonalcoholic Steatohepatitis (NASH) / Nonalcoholic Fatty Liver Disease (NAFLD) were the main causes of hospitalization (31.6% and 21.1%, respectively), followed by viral hepatitis B/C (18.4%) (Figure 1).

**Figure 1:**
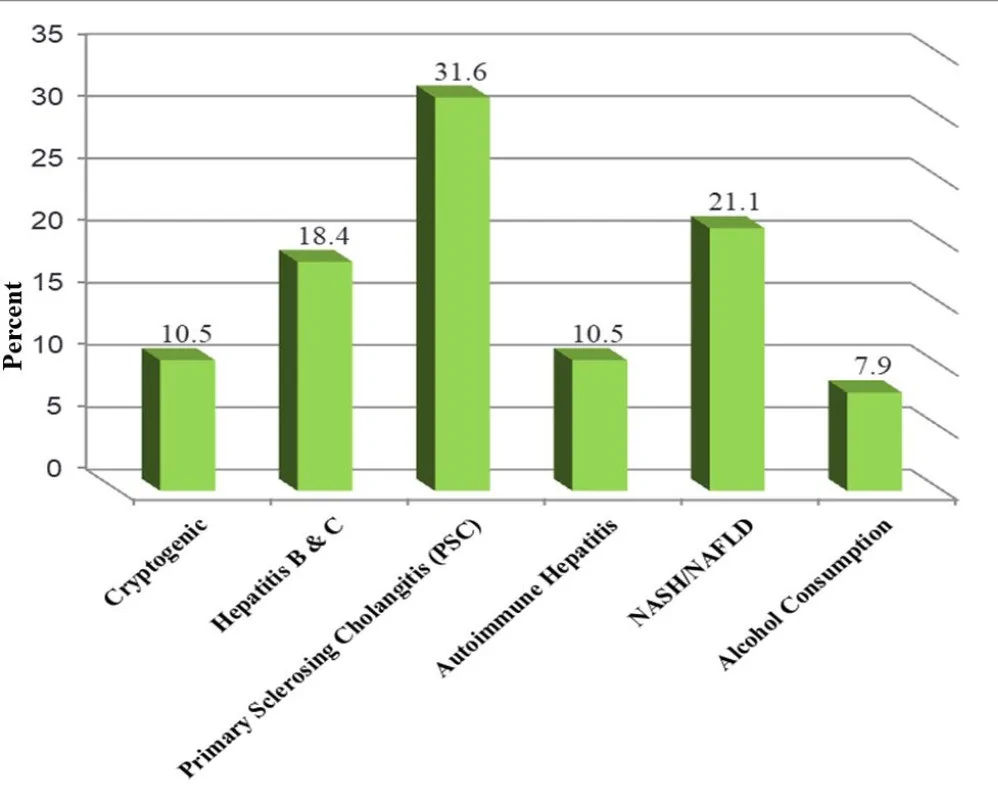
Distribution of cirrhosis etiologies among patients

Figure 2 shows the proportion of participants meeting the minimum recommended daily servings for key food groups. The adequacy of vegetable intake ( ≥ 3 servings/day) was notably lower, with only 38% of individuals reaching the threshold. For protein sources, plant-based protein intake ( ≥ 1 serving/day) was sufficient in 56.5% of the sample, while only 20.7% met the minimum suggestion for dairy consumption. When comparing food group intake adequacy between the sarcopenic and non-sarcopenic groups, the overall adequacy proportions were consistently lower in the sarcopenic group. However, except for the vegetable group (*P* = 0.03), these differences did not reach statistical significance (all *P* > 0.05).

**Figure 2:**
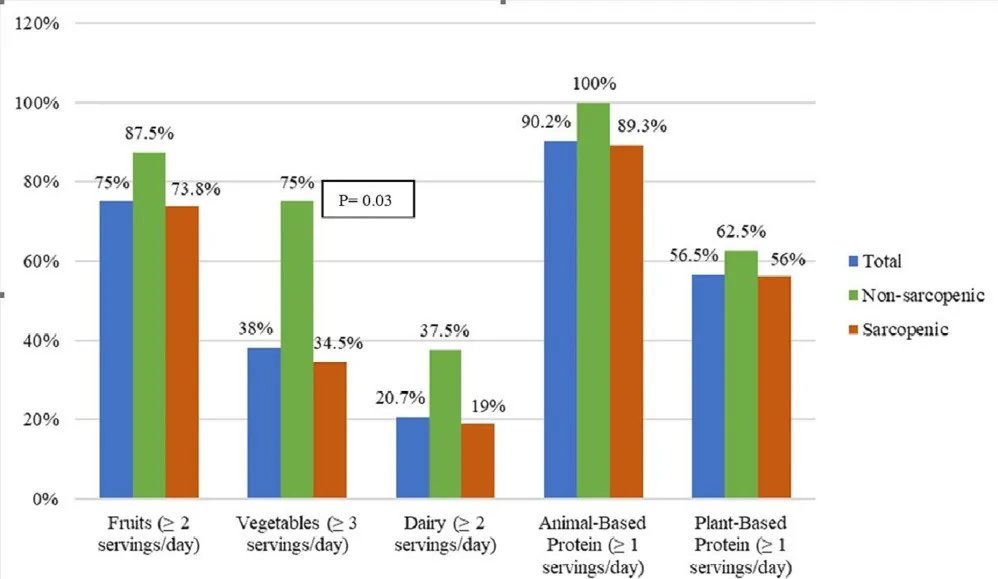
Compliance with recommended daily servings (% adequacy) of food groups in patients with cirrhosis

 Among 92 patients with liver cirrhosis (mean age: 49.1 ± 13.5 years; 57.6% men), sarcopenia was present in 66.3% of the participants. Sarcopenia was significantly linked to marital status (*P* = 0.046), showing a higher prevalence among single, divorced, or widowed individuals. Patients with sarcopenia had significantly lower body weight, BMI, waist circumference, arm circumference, and calf circumference (all *P* < 0.01). No significant differences were found in employment, education, and economic status, cause of cirrhosis, disease duration, Child-Pugh classification, or comorbidities. Functionally, patients with sarcopenia showed significantly higher rates of low muscle mass (67.2% vs. 22.6%) and impaired physical performance (98.4% vs. 64.5%) compared with non-sarcopenic patients (both *P* < 0.001, Table 3). These data clearly demonstrate the close interrelation between sarcopenia and concurrent impairments in both muscle function and quantity.

**Table 3:** Demographics, anthropometry, and clinical characteristics of all patients

**Variable**	**Total (N=92) **	**Non-sarcopenia group (n=31) **	**Sarcopenia group (n=61)**	***P*** **value**^e^
Age (years)	49.10 ± 13.45	50.45 ± 13.12	48.41 ± 13.67	0.494
Sex, n (%)				
Male	53 (57.6)	22 (71.0)	31 (50.8)	0.065
Female	39 (42.4)	9 (29.0)	30 (49.2)	
Occupation, n (%)				
Unemployed	27 (29.3)	7 (22.6)	20 (32.8)	0.595
Employee/Retired	22 (23.9)	8 (25.8)	14 (23.0)	
Self-employed	43 (46.7)	16 (51.6)	27 (44.3)	
Economic status, n (%)				
Poor	38 (41.3)	10 (32.3)	28 (45.9)	0.422
Moderate	45 (48.9)	18 (58.1)	27 (44.3)	
Good	9 (9.8)	3 (9.7)	6 (9.8)	
Marital status, n (%)				
Single/Divorced/Widowed	20 (21.7)	3 (9.7)	17 (27.9)	**0.046**
Married	72 (78.3)	28 (90.3)	44 (72.1)	
Education level, n (%)				
Illiterate	12 (13.0)	3 (9.7)	9 (14.8)	0.313
High school diploma or lower	65 (70.7)	25 (80.6)	40 (65.6)	
University degree	15 (16.3)	3 (9.7)	12 (19.7)	
Physical activity (MET)^a^, n (%)				
Low	72 (78.3)	24 (77.4)	48 (78.7)	0.889
Moderate to high	20 (21.7)	7 (22.6)	13 (21.3)	
Anthropometric indices				
Weight (kg)	72.29 ± 15.02	79.47 ± 13.32	68.65 ± 14.61	**0.001**
Height (cm)	169.47 ± 13.22	168.24 ± 16.56	170.10 ± 11.27	0.528
Body mass index (BMI)	24.81 ± 3.59	27.12 ± 3.03	23.64 ± 3.86	**<0.001**
Waist circumference (cm)	92.86 ± 12.10	97.93 ± 10.64	90.29 ± 12.06	**0.004**
Arm circumference (cm)	27.78 ± 3.76	29.89 ± 2.70	26.70 ± 3.79	**<0.001**
Calf circumference (cm)	35.49 ± 5.99	38.56 ± 7.03	33.93 ± 4.73	**<0.001**
Duration of cirrhosis (months), Median (IQR)	16.50 (43.0)	12 (31.0)	24 (49.0)	0.526
Child–Pugh classification, n (%)				
A + B	85 (92.4)	27 (31.8)	58 (95.1)	0.220
C	7 (7.6)	4 (12.9)	3 (4.9)	
Hospitalization				
No	15 (16.3)	6 (19.4)	9 (14.8)	0.572
Yes	77 (83.7)	25 (80.6)	52 (85.2)	
Presence of comorbidities, n (%)				
No	59 (64.1)	17 (54.8)	42 (68.9)	0.185
Yes	33 (35.9)	14 (45.2)	19 (31.1)	
Low muscle strength^b^, n (%)	34 (37.0)	15 (48.4)	19 (31.1)	0.105
Low muscle function ^c^, n (%)	80 (87.0)	20 (64.5)	60 (98.4)	**<0.001**
Low muscle mass^d^, n (%)	48 (52.2)	7 (22.6)	41 (67.2)	**<0.001**

**a. **Low levels of physical activity (PA): (PA) over the past 7 days < 600 (Met-Min/Week), moderate levels of (PA): (600-3000 MET- min/week) and high levels of (PA): ( > 3000 MET-min/week) due to the low percentage (2.2%) in the high levels of (PA), we combined the two levels of moderate and high to moderate to high (PA). **b. **Low muscle strength < 27 kg for men, and < 16 kg for women. **c. **A 6MWD of less than 400 m represents low muscle function. **d. **Lowmuscle mass: upper arm circumference < 27 cm for women and < 28 cm for men. **e.** Bolded values ​​are significant at the < 0.05 level. Bold numbers indicate significance

 Comparisons between the sarcopenic and non-sarcopenic groups for total energy, protein, and micronutrients are shown in Table 4. Median total energy intake was non-significantly lower in patients with sarcopenia (2547.6 kcal/day; IQR 1663.6) compared with the non-sarcopenic group (2846.6 kcal/day; IQR 2189.5, *P* = 0.435). Similarly, there was no significant difference in median absolute protein intake (100.3 g/day [IQR 73.3] vs. 105.1 g/day [IQR 71.2], *P* = 0.957) or when expressed per kg of body weight (1.47 g/kg/day vs. 1.22 g/kg/day, *P* = 0.119). The remaining micronutrient analyses also revealed no significant inter-group differences (all *P* > 0.05).

**Table 4:** Comparison of dietary and nutrient intake between the sarcopenic and the non-sarcopenic groups

**Diet components**	**Non-sarcopenia group (n=31)**	**Sarcopenia group (n=61)**	***P*** **value**^e^
Energy intake (kcal/day)	2846.64 (2189.49)	2547.62 (1663.57)	0.435
Energy intake (kcal/kg/day)	33.91 (24.90)	38.24 (23.82)	0.246
Energy deficit	78.78 (1935.58)	-197.25 (1674.83)	0.270
Protein intake (g/day)	105.15 (71.23)	100.28 (73.29)	0.957
Protein intake (g/kg/day)	1.22 (1.10)	1.47 (1.17)	0.119
Protein deficit	-1.36 (84.14)	-17.64 (76.03)	0.143
Fat intake (g/day)	87.91 (63.99)	77.31 (52.84)	0.411
Carbohydrates (g/day)	421.97 (236.18)	391.10 (224.89)	0.611
Saturated fatty acids (g/day)	30.58 (22.82)	23.94 (15.70)	0.421
Monounsaturated fatty acids (g/day)	31.51 (26.22)	27.74 (17.80)	0.416
Polyunsaturated fatty acids (g/day)	15.37 (12.61)	16.90 (12.80)	0.990
Cholesterol (mg/day)	304.31 (229.41)	278.90 (234.53)	0.918
Dietary fiber (g/day)	3.91 (4.45)	4.08 (4.12)	0.726
Sodium (mg/day)	3486.24 (3457.11)	4113.97 (2540.05)	0.379
Potassium (mg/day)	5583.66 (2748.65)	4449.47 (2409.03)	0.093
Calcium (mg/day)	1588.72 (989.76)	1129.94 (708.62)	0.091
Phosphorus (mg/day)	1990.09 (1199.46)	1667.77 (1300.04)	0.496
Magnesium (mg/day)	530.92 (229.80)	471.64 (282.92)	0.202
Iron (mg/day)	35.86 (27.14)	27.86 (19.38)	0.161
Zinc (mg/day)	17.46 (9.96)	13.71 (9.92)	0.402
Vitamin A (μg/day)	724.85 (643.22)	602.17 (528.35)	0.366
Vitamin E (mg/day)	12.82 (9.43)	11.63 (9.24)	0.440
Vitamin B1 (mg/day)	2.39 (1.94)	2.31 (1.51)	0.997
Vitamin B2 (mg/day)	2.35 (1.97)	2.12 (1.45)	0.430
Vitamin B6 (mg/day)	2.48 (1.48)	2.29 (1.68)	0.566
Vitamin B12 (mg/day)	4.67 (5.13)	4.90 (4.21)	0.997
Vitamin C (mg/day)	216.24 (162.14)	162.36 (144.07)	0.300
Folic acid (μg/day)	725.03 (500.37)	542.31 (532.85)	0.460

Data were presented as median (IQR).

## Discussion

 This cross-sectional study provides critical insights into the interplay between dietary intake, nutritional status, and sarcopenia in patients with liver cirrhosis, a condition characterized by progressive liver dysfunction and high rates of malnutrition-related complications. The findings show a very high prevalence of sarcopenia (66.3%) in the study group. This matches earlier reports that point to sarcopenia as a common and harmful issue in cirrhosis.^25,26^ There is a strong link between sarcopenia and marital status, with higher rates among single, divorced, or widowed individuals. This suggests that social factors, such as a lack of support systems, may exacerbate nutritional deficiencies and contribute to muscle loss. This observation highlights the need for comprehensive interventions that address both clinical and social aspects of cirrhosis management.^27^

 In the present study, while total energy, protein, and micronutrient intakes did not differ significantly between sarcopenic and non-sarcopenic groups, further analysis of food group adequacy provided additional insight. The proportion of patients meeting the minimum recommended daily servings for key food groups was generally suboptimal. Notably, vegetable intake adequacy was significantly lower among sarcopenic individuals, whereas differences in other food groups, including dairy and protein sources, were not statistically significant. This pattern suggests that specific dietary components, particularly low vegetable intake, may contribute to sarcopenic risk by causing micronutrient insufficiency and impairing antioxidant defense mechanisms.^28^ Similar findings have been reported in previous studies highlighting the protective effects of antioxidant- and polyphenol-rich foods against muscle catabolism and systemic inflammation in chronic liver disease.^27,29^ These results emphasize that the quality of dietary intake, rather than total caloric or protein quantity alone, may play a crucial role in modulating sarcopenic progression in patients with cirrhosis.

 The lack of significant differences in energy, protein, and micronutrient intake between sarcopenic and non-sarcopenic patients in our study, contrary to previous studies that have found a connection,^30^ may reflect the complex metabolic derangements in cirrhosis, including hypermetabolism, altered protein synthesis, and increased muscle catabolism, which can override the effects of dietary intake.^31^ Additionally, the small sample size may have limited the statistical power to detect small differences in dietary intake. This is especially true given the high variability in dietary data and the different metabolic profiles of patients with cirrhosis. The cross-sectional design of this study may also restrict the ability to observe changes in dietary patterns over time or their overall effect on muscle health.

 The etiology of cirrhosis in our study, with autoimmune hepatitis and primary sclerosing cholangitis as leading causes of hospitalization, differs from global trends, where viral hepatitis and alcoholic liver disease predominate.^32^ This variation may reflect regional differences in disease epidemiology or referral patterns to the study center. The high percentage of miscellaneous causes, at 44.6%, suggests difficulties in diagnosis and the variety of causes of cirrhosis. This could affect nutritional status and the risk of sarcopenia. For example, autoimmune-related cirrhosis may lead to systemic inflammation, which could worsen muscle wasting.^33,34^

 The low intake of important food groups is concerning. Only 38% of patients eat the recommended three servings of vegetables per day, and just 20.7% meet the dairy recommendations. This reveals a significant gap in the dietary quality of patients with cirrhosis. These findings are concerning, as vegetables provide essential micronutrients and antioxidants that may mitigate oxidative stress in liver disease, while dairy is a vital source of high-quality protein and calcium, both of which are crucial for muscle maintenance.^30,35,36^

## Implications and Future Directions

 The high prevalence of sarcopenia and suboptimal dietary intake in the current study emphasizes the urgent need for integrated nutritional and physical rehabilitation strategies in cirrhosis care. Interventions should prioritize personalized dietary plans that tackle both macronutrient and micronutrient deficiencies. These plans should work alongside resistance training to prevent muscle loss.^8,37,38,39^ Social factors, such as marital status and employment, may play a significant role, suggesting that approaches involving social workers and community support could enhance patient outcomes. Further studies with larger, more diverse groups are crucial. They will help confirm possible sex-related differences in sarcopenia prevalence and dietary habits. These studies will also clarify how the causes of cirrhosis affect nutritional needs and the risk of malnutrition.

## Conclusion

 This study highlights the high prevalence of sarcopenia in patients with liver cirrhosis and identifies critical gaps in nutritional adequacy, particularly for vegetables and dairy products. While no significant differences were observed in overall energy or protein intake, the consistently lower adequacy of food group intake among patients with sarcopenia underscores the complex, multifactorial nature of sarcopenia in this population. These findings emphasize the need for comprehensive, multidisciplinary strategies that integrate nutritional counseling, social support, and muscle-strengthening interventions to improve patient outcomes.

## Supplementary File

Table S1. Components of food groups
