# Supplementary material for: Dietary Intake, Nutritional Status, and Sarcopenia in Patients with Liver Cirrhosis: a Cross-Sectional Study
Source: Middle East J Dig Dis. 2026 Jan 31;18(1):25–33. doi: 10.34172/mejdd.3338 (PMC13680739; doi:10.34172/mejdd.3338)
Supplement: Supplementary file 1 [file mejdd-18-25-s001.pdf]

Middle East J Dig Dis 2026;18(1):25-S1
doi:10.34172/mejdd.3338
http://mejdd.org

Table S1: Components of food groups

Foods groups	Food items
Plant_based Proteins	Lentils; Beans; Peas; Chickpeas; Soybeans; Nuts and seeds
Animal_based Proteins	Beef meat & Lamb meat (tournedos, steak, mince); Hamburger; Processed meats (sausage, kielbasa);<br>Organs meats of lamb and/or beef: liver, brain, tongue;<br>Fish; poultry; canned tuna;Eggs
Dairy products	Plain semi-skimmed milk; Plain skimmed milk; Low-fat yogurts; Low-fat yogurts, soft white cheeses; Lower-fat cheeses; Plain whole milk; Flavoured milk; Natural whole yogurts; Soft rind cheeses; Dairy drinks (dough, buttermilk)
Fruits	All types of fresh fruits, dried fruits, dates, fresh fruit juices
Vegetables	All types of fresh, cooked, and dried vegetables as well as vegetable juice
